# A simple and effective method to remove pigments from heterologous secretory proteins expressed in *Pichia pastoris*

**DOI:** 10.1007/s44307-024-00013-z

**Published:** 2024-02-08

**Authors:** Tingting Li, Hongmin Cai, Yanling Lai, Hebang Yao, Dianfan Li

**Affiliations:** CAS Center for Excellence in Molecular Cell Science, Shanghai Institute of Biochemistry and Cell Biology, University of Chinese Academy of Sciences, Chinese Academy of Sciences, 320 Yueyang Road, Shanghai, 200030 China

**Keywords:** Detergent, Divalent nanobody, Lauryldimethylamine *N*-oxide, Neutralizing antibody, *Pichia* pigment, SARS-CoV-2, Secretion expression, Spike receptor-binding domain, Synthetic nanobody

## Abstract

*Pichia pastoris* is a popular yeast host for high-level heterologous expression of proteins on an industrial scale owing to its reliable expression, robust growth, high fermentation density, and easy genetic manipulation and cultivation at a relatively low cost. Of particular interest is its high secretion efficiency for small proteins including insulin, human serum albumin, vaccines, enzymes, and llama-derived heavy-chain only antibodies (nanobodies) for pharmaceutical and research applications. However, a recurring challenge in using *P. pastoris* heterologous secretory proteins is the co-purification of a sticky, yellow pigment which has been identified as a tetra-benzoyl disaccharide. Current methods for pigment removal involve crystallization of the heterologous secretory protein, active carbon absorption, and chromatography using cation exchange and hydrophobic interaction. Here, we present a simple and effective method to remove the yellow pigment, demonstrated with divalent nanobodies targeting SARS-CoV-2. The method entails capturing the nanobody on an affinity column and subsequent washing with the zwitterionic detergent lauryldimethylamine *N*-oxide (LDAO). We anticipate the method become generally useful to remove pigments from secretion proteins produced in *P. pastoris*, offering a practical solution to enhance the purity of heterologous proteins in various biotechnological applications.

## Highlights

• High-level secretion expression (~250 mg L-1) of divalent nanobodies in Pichia.

• Detergent washing effectively removes yellow pigment from secreted nanobodies.

• Nanobodies after pigment removal remain biologically active.

## Introduction

*Pichia pastoris* is a robust yeast expression system with many unique advantages (Ahmad et al. 2014; Juturu and Wu 2018). It can grow rapidly on inexpensive medium including defined medium for metabolic labeling (Matthews et al. 2018; Wood and Komives 1999). Available plasmids and kits enable convenient integration of gene-of-interest into the genome, resulting in stable strains with reliable expression (Ahmad et al. 2014); in addition, the multi-site integration and evolving technologies (Marx et al. 2009; Krainer et al. 2012) facilitate ultra-high expression level of both soluble (Sreekrishna et al. 1988; Xiong et al. 2005; Romanos 1995; Cregg et al. 1987) and membrane proteins (Cai et al. 2019; Byrne 2015).

Expression of target proteins can be driven by strong constitutive promoters (Arruda et al. 2016; Xu et al. 2018) or by methanol-inducible promoters in methylotrophic strains (Ahmad et al. 2014; Romanos 1995; Xu et al. 2018; Chang et al. 2018). Conveniently, recombinant proteins may be expressed extracellularly (Lin-Cereghino et al. 2013), bypassing the cell lysis step which is particularly challenging for yeast (Kim et al. 2013), and minimizing contamination from intracellular proteins during purification. Unlike bacterial systems, proteins purified from *Pichia* lack lipopolysaccharide (endotoxin), a costly impurity that can cause septic shock in humans for intravenous applications (Razdan et al. 2019). In addition, as a eukaryotic organism, *P. pastoris* is capable of making post-translational modifications such as glycosylation (Ahmad et al. 2014; Higgins 2001; Macauley-Patrick et al. 2005), crucial for the correct folding of some recombinant proteins. Lastly, *P. pastoris* has a unique advantage in scalable capacity, achieving extremely high cell density (OD_600_ of 500 or 120-150 g/L of biomass in fermenters) while maintaining expression levels (Cereghino and Cregg 2000). Therefore, *P. pastoris* has been a popular host for large-scale production of recombinant proteins across pharmaceutical, research, and industrial applications. These proteins include research tool proteins (Files et al. 2001; Nokelainen et al. 2001), digestive enzymes in feed and food industry (Xiong et al. 2005; Guerrero-Olazarán et al. 2010; Zhao et al. 2010; Shu et al. 2015; Cayetano-Cruz et al. 2016; Karim et al. 2019; Minning et al. 1998) such as acid phytase (Xiong et al. 2005), glucoamylase (Karim et al. 2019), lipase (Minning et al. 1998), and vaccines (Cregg et al. 1987; Farnós et al. 2005; Shukla et al. 2017), therapeutic single-chain antibodies (nanobodies) (Spadiut et al. 2014; Roy et al. 2015), and bioactive proteins (Polez et al. 2016; Radulescu et al. 2009; Nagai et al. 2003; Katla et al. 2019; Mallem et al. 2014; Bos et al. 2003; Yu et al. 2018; Rosenfeld et al. 1996) such as insulin (Polez et al. 2016), human interleukin-11 (Yu et al. 2018), and Hirudin (Rosenfeld et al. 1996).

However, a significant challenge arises in the purification of secreted proteins from *P. pastoris* due to the production of substantial amounts of pigments during methanol induction. These pigments adhere non-specifically and tightly to proteins (Li et al. 2018; Belew et al. 2008; Moore et al. 2010; Steglich et al. 2020; Azadi et al. 2018). A recent study (Moore et al. 2010) has identified the pigment as a benzoylated disaccharide (Glu1-2Xyl-N-Ac) capable of interacting with proteins through electrostatic, hydrophobic and hydrogen-bonding interactions. The removal of these pigments can be variably difficult depending on the nature of the proteins. Existing methods include hydrophobic interaction chromatography and cation exchange chromatography (Moore et al. 2010; Azadi et al. 2018), active carbon (Azadi et al. 2018), and crystallization of the target protein (Moore et al. 2010). Chromatographic methods often involve specialized machines due to the need for gradient solutions in elution, while crystallization technique is target-specific and time-consuming to develop for individual proteins.

Here, we present a simple, efficient, and effective method for pigment removal, using single-chain antibodies (nanobodies) as a case study. The method involves employing the zwitterionic detergent lauryldimethylamine *N*-oxide (LDAO) to wash off the pigment from the protein immobilized on an affinity column.

## Material and methods

Yeast nitrogen base without amino acids (YNB, Cat. 233510) was purchased from BD (Sparks, MD, USA). Zeocin (Cat. ant-zn-5b) was from InvivoGen (San Diego, CA, USA). Yeast extract (Cat. LP0021) was from Oxoid (Hampshire, UK). Peptone (Cat. 82962) was obtained from Sigma (St. Louis, MO, USA). *Sac*I restriction enzymes were purchased from Thermo Fisher Scientific (Waltham, MA, USA). Triton X-100 (Cat. X100, Lot. SLBH4329V), *N,N*-dimethyl-*N*-dodecylglycine betaine (Empigen) (Cat. 30326), lauryldimethylamine *N*-oxide (LDAO) (Cat. 40236) were sourced from Sigma. *n*-dodecyl-β-D-maltopyranoside (DDM, Cat. D310S) was purchased from Anatrace. Other chemicals were sourced from Amresco, Sigma or Sangon Bioteck (Shanghai, China). *P. pastoris* strains GS115 (*his4* Mut^+^*)* and SMD1168H (*pep4* Mut^+^) were a kind gift from Professor Rui Bao at Sichuan University, China.

### Expression of sybodies in *Pichia pastoris*

**Fig. 1:**
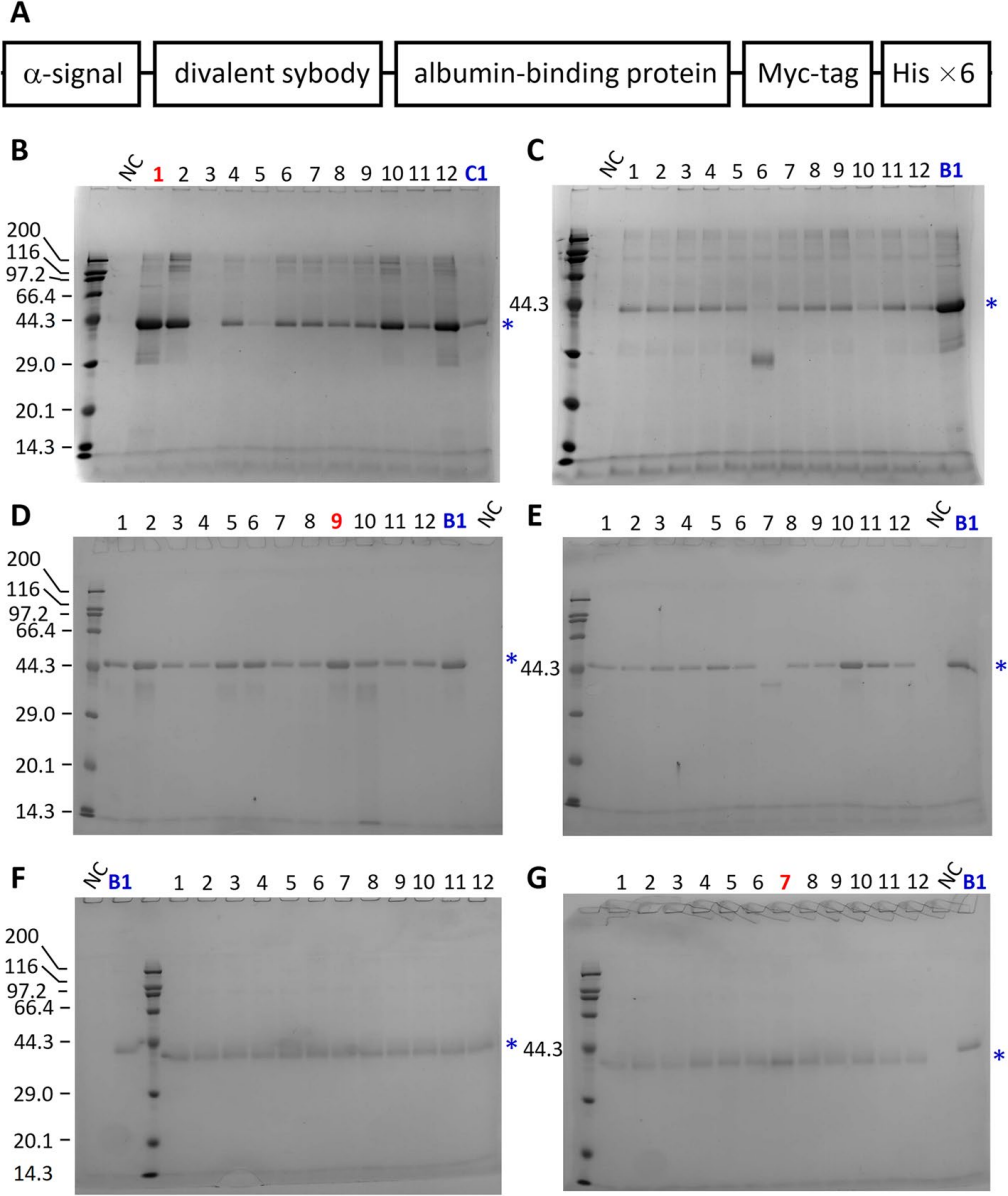
Secretion expression of sybodies in *Pichia*. **A** Schematic of the expression constructs. To avoid steric hindrance, Gly-Ser linkers (13, 20 or 34 amino-acids long) are included between individual sybodies and a 15-amino acid linker was placed between the divalent sybody and the albumin-binding domain. Detail sequence are available in ref. (Li et al. 2020). **B-G** The expression level was assessed by running 10 μL of the medium on SDS-PAGE. **B**, MR3-MR3-ABD in GS115; **C**, MR3-MR3-ABD in SMD1168H; **D**, MR17m-MR17m-ADB in GS115; **E**, MR17m-MR17m-ADB in SMD1168H; **F**, Sb44-Sb92-ABD in GS115; **G**, Sb44-Sb92-ABD in SMD1168H. Each gel contains a lane with the same protein standards with molecular weights (kDa) shown on the left (labeled for all markers in B, D, and F; and only for the 44.3-kDa band for C, E, and G). A blue star symbol indicates the band of the sybodies. Each gel contains a negative control (NC) which are non-transformed *Pichia* cells. For normalization purposes, a sample from a different group was included in each gel. In B, colony C1 (lane 1 in panel C) was included to compare the expression level of the GS115 colonies with SMD1168H colonies. Similarly, colony B1 (lane 1 in panel B) was included in C. Colony B1 was also included in gels in D-G. Colonies marked with a red number were selected for large-scale expression

DNA encoding the sybodies were cloned into the vector pPICZαC (Thermo Fisher Scientific) by Gibson Assembly. The resulting plasmids encode proteins, from the N-terminus to the C-terminus, the α-factor signal sequence, a Gly-Ser linker with sequence GSGSSS, divalent sybodies fused with the albumin binding domain (ABD) (Li et al. 2020; Yao et al. 2020), a Myc tag (EQKLISEEDL), a linker with sequence NSAVD, and a hexa-His tag (Fig. 1A, Table 1). DNA sequences were verified by DNA sequencing. The plasmids were linearized by *Sac*I digestion and transformed into *P. pastoris* GS115 and SMD1168H competent cells by electroporation in a 0.2-cm cuvette using a Bio-Rad electroporation machine (Gene Pulser Xcell) with the pre-implemented ‘Yeast’ program (Cai et al. 2019). After electroporation, cells were plated on YPDS agar plates (1 %(w/v) yeast extract, 2 %(w/v) peptone, 2 %(w/v) D-glucose, 0.8 M sorbitol, 2 %(w/v) agar) supplemented with 0.5 mg mL^-1^ zeocin. The plates were incubated at 30 °C for 2 days for colonies to grow. Colonies were first screened for expression in small-scales as follows. Colonies were inoculated into 3 mL of YPD medium (1 %(w/v) yeast extract, 2 %(w/v) peptone, 2 %(w/v) D-glucose) in a 15-mL Falcon tube and cultured overnight at 30 °C in an orbital shaker with shaking at 250 rotation per minute (r.p.m.). Cells were spun down by centrifugation at 3,000 g for 5 min at Room Temperature (RT), washed once with 2 mL BMMY (2 %(w/v) peptone, 1 %(w/v) yeast extract, 1.34 %(w/v) YNB, 400 μg L^-1^ biotin, 0.5 %(v/v) methanol, 100 mM potassium phosphate pH 5.5) and then re-suspended with 3 mL BMMY to a cell density of OD_600_ of 3-4. Cells were further grown in a shaker at 20 °C for 48 h to express sybodies. To compare the expression level, equal amounts of cell medium harvested by centrifugation were loaded onto an SDS-PAGE and the protein bands were visualized by Coomassie blue staining.

**Table 1:** Sequences of the sybody open reading frames for secretion expression in *P. pastoris*

**Sybody**	**Sequence**
MR3-MR3-ABD	MRFPSIFTAVLFAASSALAAPVNTTTEDETAQIPAEAVIGYSDLEGDFDVAVLPFSNSTNNGLLFINTTIASIAAKEEGVSLEKREAEAGSGSSSQVQLVESGGGLVQAGGSLRLSCAASGFPVNAHFMYWYRQAPGKEREWVAAIYSYGRTLYADSVKGRFTISRDNAKNTVYLQMNSLKPEDTAVYYCNVKDYGAASWEYDYWGQGTQVTVS*GGGGSGGGGSGGGGSGGGGSGGGGSGGGGSGSSS*QVQLVESGGGLVQAGGSLRLSCAASGFPVNAHFMYWYRQAPGKEREWVAAIYSYGRTLYADSVKGRFTISRDNAKNTVYLQMNSLKPEDTAVYYCNVKDYGAASWEYDYWGQGTQVTVSAGRAG*GGGGSGGGGSGGGGS*GTIDEWLLKEAKEKAIEELKKAGITSDYYFDLINKAKTVEGVNALKDEILKAEQKLISEEDLNSAVDHHHHHH*
MR17m-MR17m-ABD	MRFPSIFTAVLFAASSALAAPVNTTTEDETAQIPAEAVIGYSDLEGDFDVAVLPFSNSTNNGLLFINTTIASIAAKEEGVSLEKREAEAGSGSSSQVQLVESGGGLVQAGGSLRLSCAASGFPVEVWRMEWYRQAPGKEREGVAAIESYGHGTRYADSVKGRFTISRDNAKNTVYLQMNSLKPEDTAVYYCNVYDDGQLAYHYDYWGQGTQVTVS*GGGGSGGGSGSSS*QVQLVESGGGLVQAGGSLRLSCAASGFPVEVWRMEWYRQAPGKEREGVAAIESYGHGTRYADSVKGRFTISRDNAKNTVYLQMNSLKPEDTAVYYCNVYDDGQLAYHYDYWGQGTQVTVSAGRAG*GGGGSGGGGSGGGGS*GTIDEWLLKEAKEKAIEELKKAGITSDYYFDLINKAKTVEGVNALKDEILKAEQKLISEEDLNSAVDHHHHHH*
Sb44-Sb92-ABD	MRFPSIFTAVLFAASSALAAPVNTTTEDETAQIPAEAVIGYSDLEGDFDVAVLPFSNSTNNGLLFINTTIASIAAKEEGVSLEKREAEAGSGSSSQVQLVESGGGLVQAGGSLRLSCAASGFPVDTQWMHWYRQAPGKEREWVAAISSTGRSTFYADSVKGRFTISRDNAKNTVYLQMNSLKPEDTAVYYCTVYVGNRYRGQGTQVTVS*GGGGSGGGGSGGGGSGGGGS*QVQLVESGGGLVQAGGSLRLSCAASGFPVGRASMWWYRQAPGKEREWVAAISSYGWVTAYADSVKGRFTISRDNAKNTVYLQMNSLKPEDTAVYYCEVSVGTGYRGQGTQVTVSAGRAG*GGGGSGGGGSGGGGS*GTIDEWLLKEAKEKAIEELKKAGITSDYYFDLINKAKTVEGVNALKDEILKAEQKLISEEDLNSAVDHHHHHH*

For large-scale expression, the colony with the highest expression level of sybodies was chosen. The procedure was the same as for the small-scale screening except that the volume at each step was scaled to suit the final protein production in 3-L flasks. In detail, 10-mL overnight cell culture were inoculated into 1-L YPD in a 3-L flask, and cultured overnight at 30 °C in an orbital shaker with shaking at 250 r.p.m.. Cells were spun down by centrifuging at 4,000 g for 10 min at RT, washed with 100 mL BMMY and then resuspended with 100 mL BMMY to increase the OD_600_ to 100, shake at 20 °C for 4-5 days for induction. For each 24 h, 5 %(v/v) methanol was added to induce the expression. The medium was collected by centrifugation at 5,000 g, filtered through 0.22-μm membrane and the supernatant was used for purification immediately.

### Dialysis

*Pichia* medium (in 0.35-L batches) was placed in a 14-kDa cut-off dialysis membrane (Cat. D9402, Sigma) for dialysis at 20 °C against 5.5 L of 1 × PBS buffer (1.76 mM KH_2_PO_4_, 8.8 mM Na_2_HPO_4_, 2.68 mM KCl, 137 mM NaCl) with mild agitation. The dialysis was repeated three times (typically 6-h, 6-h, and 16-h) with a fresh dialysis buffer between each time.

### Ammonium sulfate precipitation

To 0.35-L yeast medium (without dialysis), solid ammonium sulfate (Cat. A501076-0500, Sangon Biotech) was added slowly and with mild agitation to 24.2 %(w/v). The solution was placed at 4 °C for 4 h before centrifugation at 30,000 g for 30 min at 4 °C. The pellet containing the sybodies was re-dissolved in 50 mL of 1 × PBS buffer for further purification.

### Protein A-affinity chromatography

Sybodies from ammonium sulfate precipitation were dialyzed against 4 L of 1 × PBS buffer supplemented with 1 mM EDTA for three times and then remove the insoluble materials at 30,000 g for 30 min at 4 °C. The supernatant was incubated with Protein A resin (Cat. SA012005, Smart-lifesciences) at 4 °C for 1 h. After brief washing (the sybodies only bind to Protein A beads weakly), the sybodies were eluted using 0.1 M glycine pH 3.0 and then immediately neutralize with by adding a tenth volume of 1.5 M NaCl, 1 M Tris HCl pH 8.0.

### Immobilized metal affinity chromatography

Sybodies from the dialyzed medium, or as in the elution of the Protein A beads, were incubated with Ni-NTA beads (Cat. 1018401, Qiagen) for immobilized metal affinity chromatography (IMAC). Typically, 1 mL of Ni-NTA beads for every 0.1 L of culture was used. After 2 h of batch-binding at 4 °C, the beads were placed in a gravity column. The column was washed with 20 column volume (CV) of 20 mM imidazole before eluted with 300 mM imidazole in Buffer A (150 mM NaCl, 20 mM Tris pH 8.0).

### Detergent washing

Sybodies immobilized on the IMAC column were first washed with 20 mM imidazole as described above. Detergents (Triton X-100, DDM, LDAO, Empigen) were individually prepared as 1% solution in Buffer A. The beads were washed with 20 CV of 1% detergents in Buffer A, followed by 30 CV of Buffer A. In the case of LDAO, 0.1 % and 0.5 % of LDAO in Buffer A were additionally tested by washing the resin for 20 CV. Protein was eluted from the beads using 300 mM imidazole in Buffer A.

### Binding assay

Biolayer interferometry (BLI) binding assays were carried out using an Octet RED96 system (FortéBio). Biotinylated RBD (Li et al. 2020) was immobilized on a streptavidin sensor (Cat. 18-5019) by incubating the sensor in 2 μg mL^-1^ of RBD in the Binding Buffer (0.005 %(v/v) Tween 20, 150 mM NaCl, 20 mM Tris HCl pH 8.0) at 30 °C. The sensor was then equilibrated in the RBD-free Binding Buffer (baseline) for 330 s, before incubating with MR3-MR3-ABD at 10 nM (association) for 300 s. The sensor was then incubated with sybody-free Binding Buffer for dissociation and the BLI signal was monitored for another 300 s. MR3-MR3-ABD expressed in *E. coli* was purified as previously reported (Li et al. 2020).

### Active carbon treatment

The *Pichia* medium containing secreted sybodies were incubated with 1%, 2%, 3%, and 5% active carbon in an Eppendorf tube. After incubation at 4 °C for 1.5 h, the mixture was centrifuged at 20,000 g for 20 min at 4 °C. The supernatant fractions at equal volume (10 μL), together with a control sample without treatment, were analyzed by SDS-PAGE and visualized by Coomassie staining.

### Spectroscopy and protein quantification

Absorbance spectroscopy was performed using a Nanodrop machine by applying 2 μL of the sample directly onto the machine. Purified sybodies were quantified using absorbance at 280 nm acquired with a Nanodrop machine and the molar extinction coefficient of 85,510 M^-1^ cm^-1^ (MR3-MR3-ABD), 71530 M^-1^ cm^-1^ (MR17m-MR17m-ABD), and 68,090 M^-1^ cm^-1^ (Sb44-Sb92-ABD).

## Results and discussion

### High-level expression of sybodies in *Pichia pastoris*

Previously (Li et al. 2020; Yao et al. 2020; Li et al. 2022 Li et al. 2022), we identified several nanobodies and synthetic nanobodies (sybodies) against the Spike receptor-binding domain (RBD) of SARS-CoV-2, the causative virus of Covid-19. Some of the sybodies bind the RBD at a site that was required for the interaction between RBD and the host cell receptor ACE2, and therefore inhibits the viral infection when tested using pseudoviruses. To test their potency in mice and hamsters, large quantities (dozens of milligrams) were required (Li et al. 2020; Li et al. 2021).

To avoid the problem of endotoxin, we chose the *Pichia* system for expression. The sybodies were specially engineered as follows. First, they were made divalent by direct fusion with a Gly-Ser linker that contains 34, 20 or 13 amino-acids. Second, the albumin-binding domain (ABD), together with a 15-amino-acids Gly/Ser linker, was fused to the C-terminus of the divalent sybodies for prolonged half-lives in vivo (Li et al. 2020) (Fig. 1A, Table 1). Three sybodies were used in this study. MR3-MR3-ABD (39.2 kDa) and MR17m-MR17m-ABD (38.3 kDa) were neutralizing sybodies. The non-neutralizing sybody (negative control for animal experiments), Sb92-Sb44-ABD (36.7 kDa), was a biparatopic antibody against a thermostable green fluorescence protein (Cai et al. 2020). Because the three sybodies behaved similarly during purification, we’ll universally refer them to as sybody in this study unless specified.

The sybodies were expressed in two *P. pastoris* strains (GS115 and SMD1168H) with an α-factor secretion signal at the N-terminus (Fig. 1A, Table 1). Therefore, they could be detected conveniently by analyzing the medium on SDS-PAGE. Because the expression level of the target protein in *Pichi*a depends on the position and copy number of target gene integrated into the genome, we first screened colonies for their expression level. As shown in Fig. 1B-G, most colonies showed a band at the expected position of the sybodies on SDS-PAGE and the band was not present in the negative control which are untransformed cells. For MR3-MR3-ABD, one colony, GS115 B3 (panel B, lane 3) did not show expression (Fig. 1B), and SMD1168H C6 showed a band that ran faster than expected (Fig. 1C), suggesting degradation in this colony. Similarly, the Colony E7 for MR17m-MR17m-ABD (Fig. 1E) also showed a band with lower apparent molecular weight than others. In most cases, the samples had a purity of greater than 70%. Based on the expression level, colony #B1 (MR3-MR3-ABD), #D9 (MR17m-MR17m-ABD), and #G7 (Sb92-Sb44-ABD) were selected for large-scale protein production. In a separate study, we quantified the expression level of the MR3-MR3-ABD colony B1 as 250 mg L^-1^ using SDS PAGE analysis. Specifically, the supernatant of the cell culture was loaded together with known amounts of purified MR3-MR3-ABD which had been pre-mixtured with the medium from the untransformed GS115 cell culture. The band intensity was then semi-quantified by densitometry analysis (Li et al. 2020).

The results indicate that the divalent sybodies can be expressed in *Pichia* as secreted proteins at a high-level, and highlight the importance to carry out small-scale screening for *Pichia* expression.

### Sybodies expressed in *Pichia* were bound with pigments

**Fig. 2:**
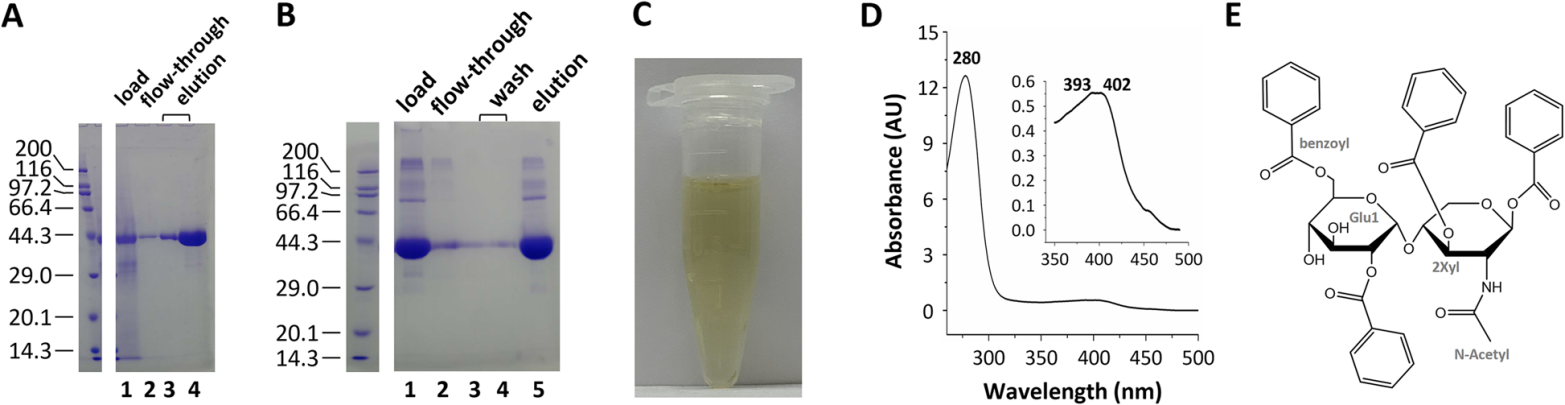
Sybodies purified from *Pichia* are contaminated with yellow pigment. **A** Purification of sybodies by immobilized metal affinity chromatography (IMAC). Lane 1, sybody after dialysis; lane 2, the flow-through fraction; lane 3 and 4, the elution fraction. The molecular weight of SDS-PAGE standards is shown on the left. **B** Pre-purification by protein A affinity chromatography enables IMAC purification of sybodies. The molecular weight of protein standards is shown on the left. Lane 1, the elution from protein A affinity chromatography; lane 2, the flow-through fraction after binding with Ni-NTA; lane 3 and 4, the wash fraction; lane 5, the elution fraction of the Ni-NTA column. **C** Image of a typical elution fraction from IMAC using protocols in A or B. **D** A typical UV-Vis spectrum of the elution fraction from IMAC using protocols in A or B. **E** Chemical structure of the benzoyl disaccharide identified in the literature (Moore et al. 2010)

Next, we set to purify the sybodies using immobilized metal affinity chromatography (IMAC). For efficient Ni-NTA binding (Fig. 2A), the medium was dialyzed against 1 × PBS buffer before incubated with Ni-NTA beads. For large-scale purification, dialysis becomes impractical. Therefore, alternatively, the medium was treated with 24.2 %(w/v) ammonium sulfate to precipitate sybodies. The re-dissolved sybody could only bind Ni-NTA beads weakly. Thus, an affinity purification step using Protein A was added. Sybodies eluted from the Protein A beads could efficiently bind to Ni-NTA (Fig. 2B). On SDS-PAGE, sybody purified in this manner had an estimated purity of >90%. However, the protein solution had a strong yellow color (Fig. 2C) even after the ammonium sulfate precipitation and affinity purification steps. In addition, the color could not be removed by anion exchange chromatography, or by washing with high salt (1 M NaCl) or metal ions (1 mM Ca^2+^ or 5 mM Mg^2+^) on Ni-NTA column. The spectrophotometric analysis revealed a broad peak in the visible wavelength with λ_max_ near 400 nm in addition to the protein peak at 280 nm (Fig. 2D). The behavior of the pigment, including its stickiness to protein during chromatography and ammonium sulfate precipitation, and the color, is consistent with what has been previously described in the literature (Li et al. 2018; Belew et al. 2008; Moore et al. 2010; Steglich et al. 2020; Azadi et al. 2018). A decade ago, the van der Walle group (Moore et al. 2010) identified the pigment as benzoylated Glu1-2Xyl-N-Ac (Fig. 2E). This compound contains four benzoyl groups which, together with the greasy side of the disaccharide, could form hydrophobic interactions with proteins. In addition, the compound is rich in carbonyl groups which accumulatively endow the molecule with the ability to attract positively-charged residues or surfaces. Finally, the abundant hydroxyl and ether groups can interact with proteins via hydrogen bonds.

### A simple wash step removed the pigment from sybodies

**Fig. 3:**
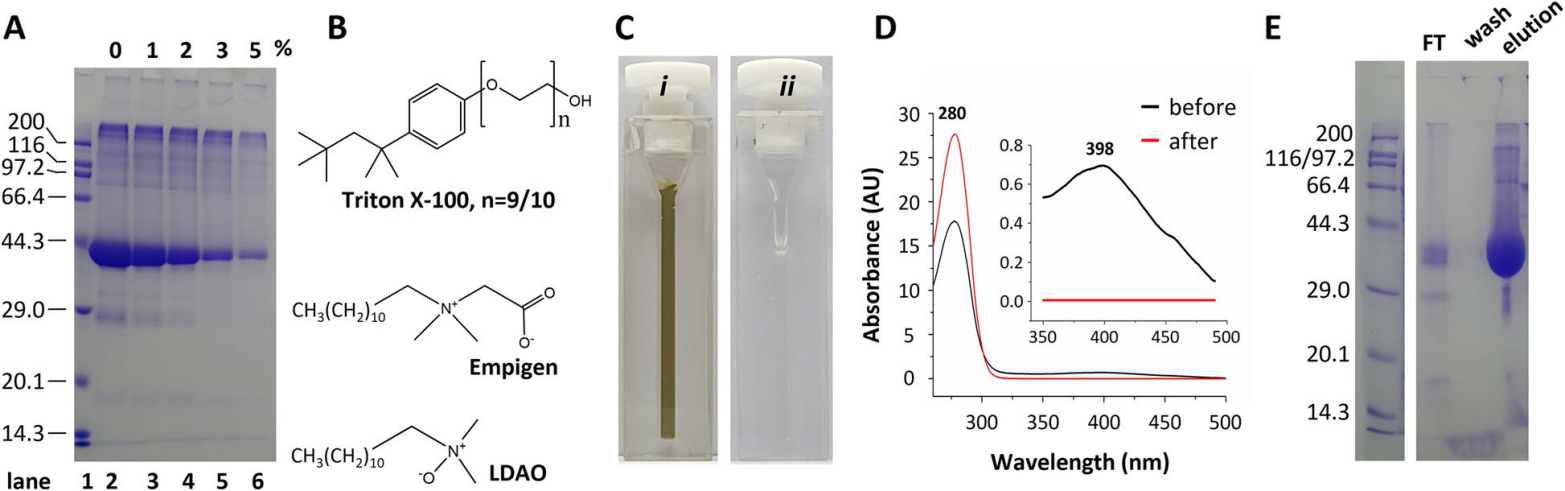
Removal of yellow pigment from sybodies purified from *Pichia*.** A** Active carbon absorbs sybodies. Equal volume of sybody samples treated with active carbon at concentrations indicated above each lane were loaded onto SDS-PAGE for electrophoresis and Coomassie staining. Lane 1, molecular marker; Lane 2, sybody sample without active carbon incubation (0%); lane 3-6, sybody samples treated with active carbon (1% - 5%). **B** Chemical structures of the detergents Triton X-100, Empigen, and lauryldimethylamine *N*-oxide (LDAO). **C** Images of the sybody sample before (*i*) and after (*ii*) LDAO wash. Samples were placed in a 1-cm path length quartz cuvette. **D** UV-Vis spectrum of the sybody sample before (black) and after (red) washing with LDAO. The inset shows the expanded view in the visible wavelength to highlight the characteristics of the yellow pigment. (**E**) A typical result of LDAO wash for the purification of divalent sybodies. The fractions of flowthrough (FT), LDAO wash (wash), and elution were analyzed by SDS-PAGE. Molecular standards were indicated on the left

Next, we sought simple methods to remove the pigment. It has been reported in the literature that active carbon can effectively absorb the pigment without significant loss of protein in the case of human growth hormone (Azadi et al. 2018). When tested for the sybodies, however, we found that the color was not completely removed even in the presence of 5%(w/v) active carbon. Rather, approximately 90% of the sybodies were lost after the treatment (Fig. 3A).

Cation exchange and hydrophobic interaction chromatography (Moore et al. 2010; Azadi et al. 2018) have been successfully used in the literature to remove the pigment. Because the sybodies have calculated isoelectric points near pH 5.0 (MR3-MR3-ABD, 5.24; MR17m-MR17m-ABD, 5.03; Sb44-Sb92-ABD, 5.63), cation exchange was not considered, as they would be overall negatively charged under our purification conditions (pH 8.0). Rather, we hypothesized that the binding between the pigment and sybodies may be disrupted competitively by zwitterionic detergents which bear both hydrophobicity and charge. Specifically, the hydrocarbon portion of detergents would compete with the benzoyl moiety of the pigment while the charged head group would disrupt hydrogen bonding between the carbonyl or the hydroxyl of the pigment and proteins. Two zwitterionic detergents, *N,N*-dimethyl-*N*-dodecylglycine betaine (Empigen), and lauryldimethylamine *N*-oxide (LDAO), and the non-ionic detergent Triton X-100 (Fig. 3B) were tested.

Triton X-100 had no effect on removing the pigment. Empigen was modestly effective. Remarkably, LDAO wash resulted in a colorless protein solution, as viewed by the naked eye (Fig. 3C) or by spectrophotometry analysis (Fig. 3D). Thus, the LDAO wash resulted in no detectable absorbance in the region between 350 and 500 nm. Further, by contrast to the active carbon method (Fig. 3A), the LDAO wash did not cause noticeable loss of sybodies (Fig. 3E).

### The wash procedure did not affect the sybody’s binding property

We therefore performed large-scale purification by adding an LDAO wash step on Ni-NTA column. After the de-coloring step, the nanobodies were washed extensively using 30 column volume of LDAO-free buffer to minimize LDAO level. We would note that the residual level of LDAO in the final product was not quantified.

**Fig. 4:**
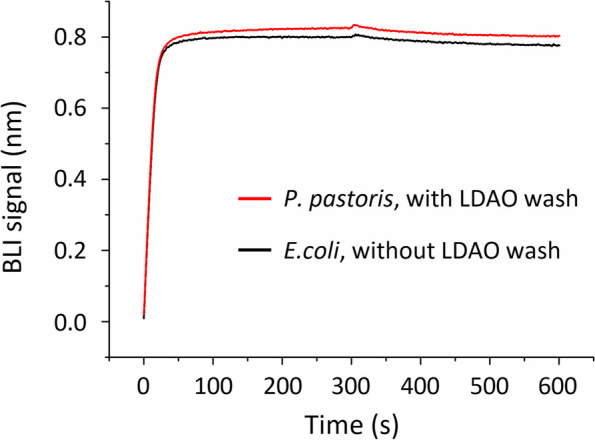
The detergent wash step did not affect binding kinetics of MR3-MR3-ABD for interaction with SARS-CoV-2 RBD. Biolayer interferometry (BLI) assays were performed using an Octet system with RBD immobilized and the sybody MR3-MR3-ABD as analyte at 10 nM concentration. Both association and dissociation were monitored for 300 s. The sybody purified from *E. coli* (black) showed a similar curve with that purified from *P. pastoris* (red). LDAO, lauryldimethylamine *N*-oxide

The binding characteristics between the sybody MR3-MR3-ABD and SARS-CoV-2 S RBD were tested using the sybody washed LDAO and compared with that from *E. coli* which was purified by IMAC without LDAO wash. As shown in Fig. 4, they showed almost identical binding curves. As we reported separately (Li et al. 2020), the sybody purified by LDAO wash could neutralize SARS-CoV-2 pseudovirus and protected mice from live virus challenge without showing noticeable toxicity.

### A recommended flowchart for the purification of nanobodies in *Pichia*

**Fig. 5:**
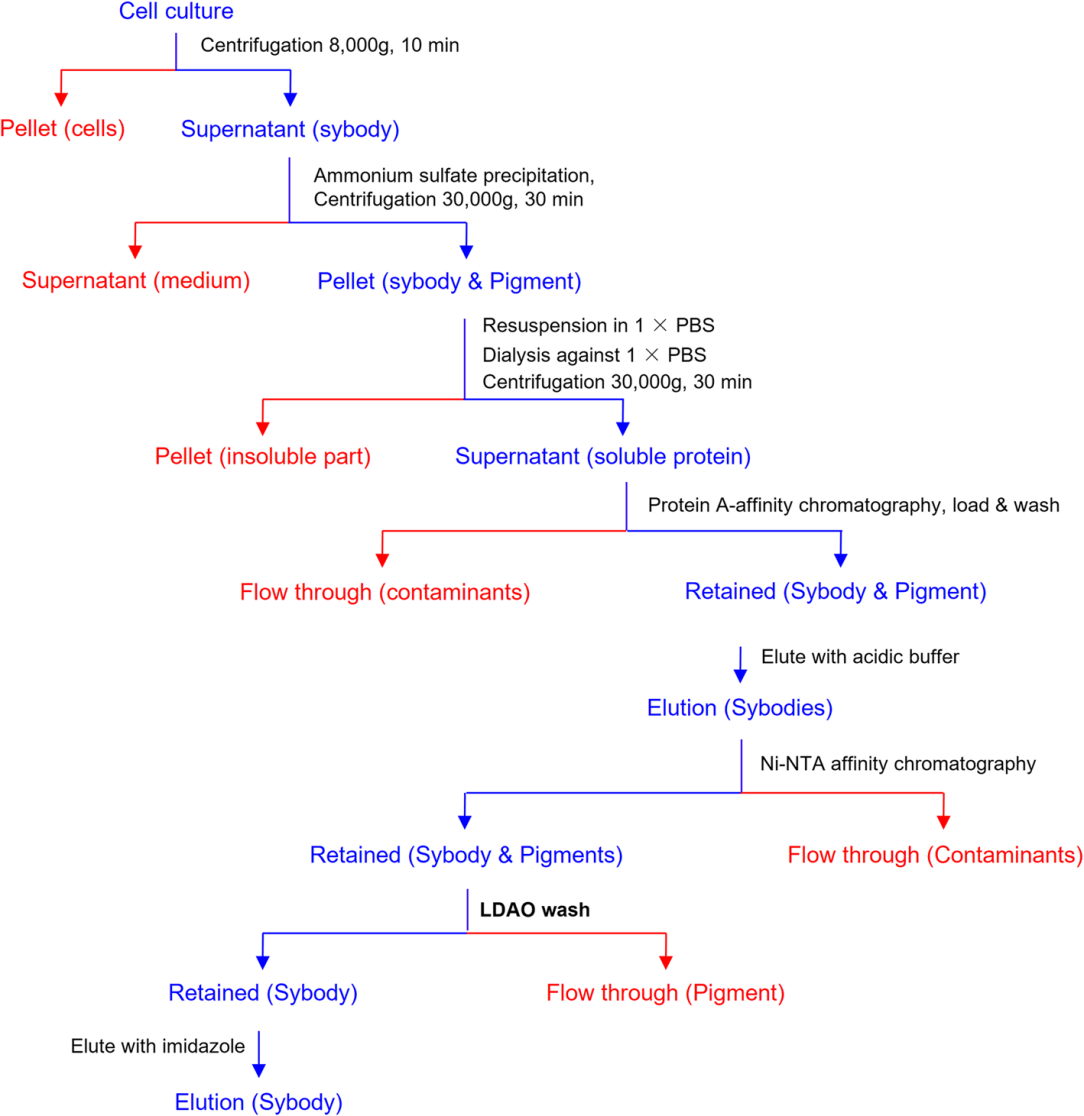
Flowchart for purification of the nanobodies secretion expressed in *Pichia*. The common steps included ammonium sulfate precipitation, dialysis, affinity purification with Protein A beads and Ni-NTA resins and a special detergent washing step to remove pigments simply are organized for the purification of nanobodies

Based on the results, we propose a flowchart for purification of nanobodies as in Fig. 5.

## Discussion

*Pichia pastoris* is a robust microorganism for production of recombinant protein at an industrial scale owing to the simplicity and cost-effectiveness in its genetic manipulation and culturing. A well-documented issue, however, is the co-purification of a yellow pigment that is difficult and costly to remove. In this work, we developed a simple, convenient, high-yield, and cost-efficient method for removing the pigment from a SARS-CoV-2-neutralizing nanobody by introducing a washing step with a zwitterionic detergent.

Compared with previous cation exchange and hydrophobic interaction chromatography methods, the detergent wash method has several advantages. First, it can be easily incorporated into affinity purification procedures since LDAO does not interfere with affinity interactions. Second, the materials introduced (LDAO) during the detergent wash can be immediately and easily removed on the same affinity column. This is by sharp contrast with the other two methods where salts are inevitably introduced during the wash, and the salts need to be removed by a separate step such as gel filtration or dialysis. Third, the detergent wash can be done on a gravity column, avoiding the use of automated equipment. This can be especially useful in laboratories where resources are limited or not easily implemented. Our work therefore provided a promising strategy to produce SARS-CoV-2 neutralizing nanobodies in large quantities. Although the general applicability of the method to other heterologous secretory proteins remains to be investigated, the non-specificity nature of the pigment-protein interaction would certainly encourage the test of this method to other proteins. In long term, the results may inspire novel application of the current or improved format(s) to the production of other bioactive proteins in both academy and industry research fields.

## Conclusions

We have developed a technique to eliminate an acidic, yellow pigment associated with secreted proteins in *Pichia*. The central aspect of this method involves washing the protein on a matrix using the zwitterionic detergent LADO. As illustrated by the purification of a SARS-CoV-2 neutralizing nanobody, proteins obtained through this protocol retain their biological activity. This method holds potential for purifying pharmaceutically relevant proteins from *Pichia*, catering to both research and industrial applications.

## Data Availability

Data are contained within the article.
